# SOX Transcription Factors in Endothelial Differentiation and Endothelial-Mesenchymal Transitions

**DOI:** 10.3389/fcvm.2019.00030

**Published:** 2019-03-28

**Authors:** Yucheng Yao, Jiayi Yao, Kristina I. Boström

**Affiliations:** ^1^Division of Cardiology, David Geffen School of Medicine at UCLA, Los Angeles, CA, United States; ^2^Molecular Biology Institute, UCLA, Los Angeles, CA, United States

**Keywords:** vascular calcification, sex determining region y-box, endothelial-mesenchymal transition, endothelium, differentiation

## Abstract

The SRY (Sex Determining Region Y)-related HMG box of DNA binding proteins, referred to as SOX transcription factors, were first identified as critical regulators of male sex determination but are now known to play an important role in vascular development and disease. SOX7, 17, and 18 are essential in endothelial differentiation and SOX2 has emerged as an essential mediator of endothelial-mesenchymal transitions (EndMTs), a mechanism that enables the endothelium to contribute cells with abnormal cell differentiation to vascular disease such as calcific vasculopathy. In the following paper, we review published information on the SOX transcription factors in endothelial differentiation and hypothesize that SOX2 acts as a mediator of EndMTs that contribute to vascular calcification.

## Introduction

Appropriate endothelial cell (EC) differentiation is essential to support vascularization of tissues and maintain proper vascular homeostasis. In coordination with tissue development, ECs are derived from progenitor cells that undergo endothelial lineage differentiation to form functional vascular networks (1, 2). In fully developed tissues, quiescent endothelium can be converted to active endothelium as needed for tissue regeneration or repair, and mature endothelial lineage is required to return to and maintain normal vasculature (3). Disease often borrows elements from development such as excessive production of morphogenic factors (4), dysregulation of stem cells (5), abnormal angiogenesis (6), and ectopic cell differentiation (7). Albeit a normal process in neural crest development and cardiac valves and neovascularization (8, 9), endothelial-mesenchymal transitions (EndMTs) contribute to vascular disease when the transitions re-emerge in atypical locations (10–16). EndMTs have been revealed as novel sources of calcifying cells for vascular calcification, which is considered to be a form of ectopic bone formation and involves multipotent cells and networks of growth factors and transcription factors (10). The SOX transcription factors have been shown to be essential mediators in vascular development. Here, we review the SOX factors in endothelial differentiation and EndMTs and include some of our results to support previous studies. In addition, we briefly review the SOX factors in EndMTs, and argue that SOX2 induces EndMTs and serves as a novel cellular source in vascular calcification.

## Vascular SOX Transcription Factors

The sex-determining region of the Y-chromosome, the SRY gene, was initially discovered as a testis-determining gene in human and mice (17, 18). It led to the discovery of the family of SRY (Sex Determining Region Y)-related HMG box of DNA binding proteins, referred to as SOX transcription factors, which consists of more than 20 *Sox* genes (19).

The SOX transcription factors are characterized by the evolutionarily conserved high mobility group (HMG) box, a 79-amino-acid DNA-binding motif that binds to a common consensus site with variable efficiency (19) and are regulated by multiple signaling pathways during vascular development (19–21). They are subdivided into the A-J groups based on phylogenetic analysis of the HMG box sequences, protein structure, gene organization, and function within developmental programs (19, 22, 23). The SOX transcription factors that have been associated with the vasculature are SOX7, SOX17, and SOX18, members of the SOXF subgroup that appear in vascular developmental programs (19–21), and SOX2, a member of the SOXB subgroup that primarily has been associated with EndMTs.

## SOX7, SOX17, and SOX18

SOX7, SOX17, and SOX18 are active in early vasculogenesis at the onset of endothelial differentiation and function upstream of signaling cascades that regulate cell fate decisions (19–21). Already on embryonic day (E) 7.5 in mice, ETV2+/FLK1+/CD41– cell populations enriched for endothelial progenitor cells show SOX7 expression in 97%, SOX18 expression in 50%, and SOX17 expression in 75% of the cells (21). SOX7 and SOX18 continue to be expressed in the dorsal aorta, cardinal vein and intersomitic vessels by E8.25 and throughout the developing vascular network at later dates (21, 24, 25). SOX17, on the other hand is detected in EphrinB2+ arterial cells by E10.5, suggesting arterial specificity already at this stage (21, 26, 27). Post-natally, SOX7 and SOX18 continue to be expressed in both arterial and venous endothelium in mice, whereas SOX17 expression is restricted to the arterial endothelium (28). Studies in zebrafish have confirmed similar roles of Sox7 and Sox18 in vascular regulation [reviewed in (21)], whereas Sox17 is not expressed in the developing vasculature of zebrafish.

SOX7 haploinsufficiency has been linked to cardiac defects and congenital diaphragmatic hernia, and is characterized by microdeletions at 8p23.1 that include the Sox7 gene (29). Global gene deletion of *Sox7* in mice is associated with embryonic lethality due to absence of the major vessels in the yolk sac and cardiovascular failure (29). Global loss of *Sox17* results in depletion of the definitive endoderm and early embryonic lethality (30). However, the cardiovascular defects in *Sox17*^−/−^ mice are more pronounced in mice with combined loss of *Sox17* and *Sox18* (24), suggesting redundancy between these two factors. Conditional endothelial-specific *Sox17* deletion using *Tie2*-Cre mice results in blocks in the vascular remodeling of the yolk sac, absence of arteries, and fusion between the aorta and the cardinal vein associated with loss of arteriovenous identity (21, 27). It implies a connection between the SOX transcription factors and Notch signaling that recognizes the important role of Notch in arteriovenous differentiation (20). It has also been shown that Notch signaling can suppress endothelial SOX17, and that this repression induces venous genes such as CoupTFII, while suppressing arterial genes such as *EphrinB2, Notch4*, and *Delta-like ligand (Dll*)4 (20, 31). SOX17 may also be a mediator of canonical Wnt signaling in arterial differentiation (32). Global *Sox18*^−/−^ mice on 129/Sv-CD1 mixed genetic background were initially reported to be viable without gross abnormalities in the cardiovascular system (33). However, *Sox18*^−/−^ mice on pure C57BL/6 background develop subcutaneous edema and embryonic lethality due to interference in the lymphangiogenesis (34, 35), which supports the hypothesis that SOX18 plays an important role in lymphangiogenesis (21, 34, 35).

## SOX2

SOX2 is essential for regulation of interactions between the epithelium and the mesenchyme (36), differentiation of multiple cell lineages (37–40) and cell fate transitions (41, 42). SOX2 may be best known as one of the four original pluripotent factors that together with octamer-binding transcription factor 3/4 (Oct3/4), Kruppel-like factor 4 (Klf4), and c-Myc is used for the reprogramming of cells (43) and serves as a marker of neural stem cells (44, 45). SOX2 also enhances the reprogramming capacity of cardiovascular cells, and has been shown to induce endothelial differentiation in isolated adult mesoangioblasts (46) and participate in the reprogramming of corneal endothelial cells (47).

**Figure 1:**
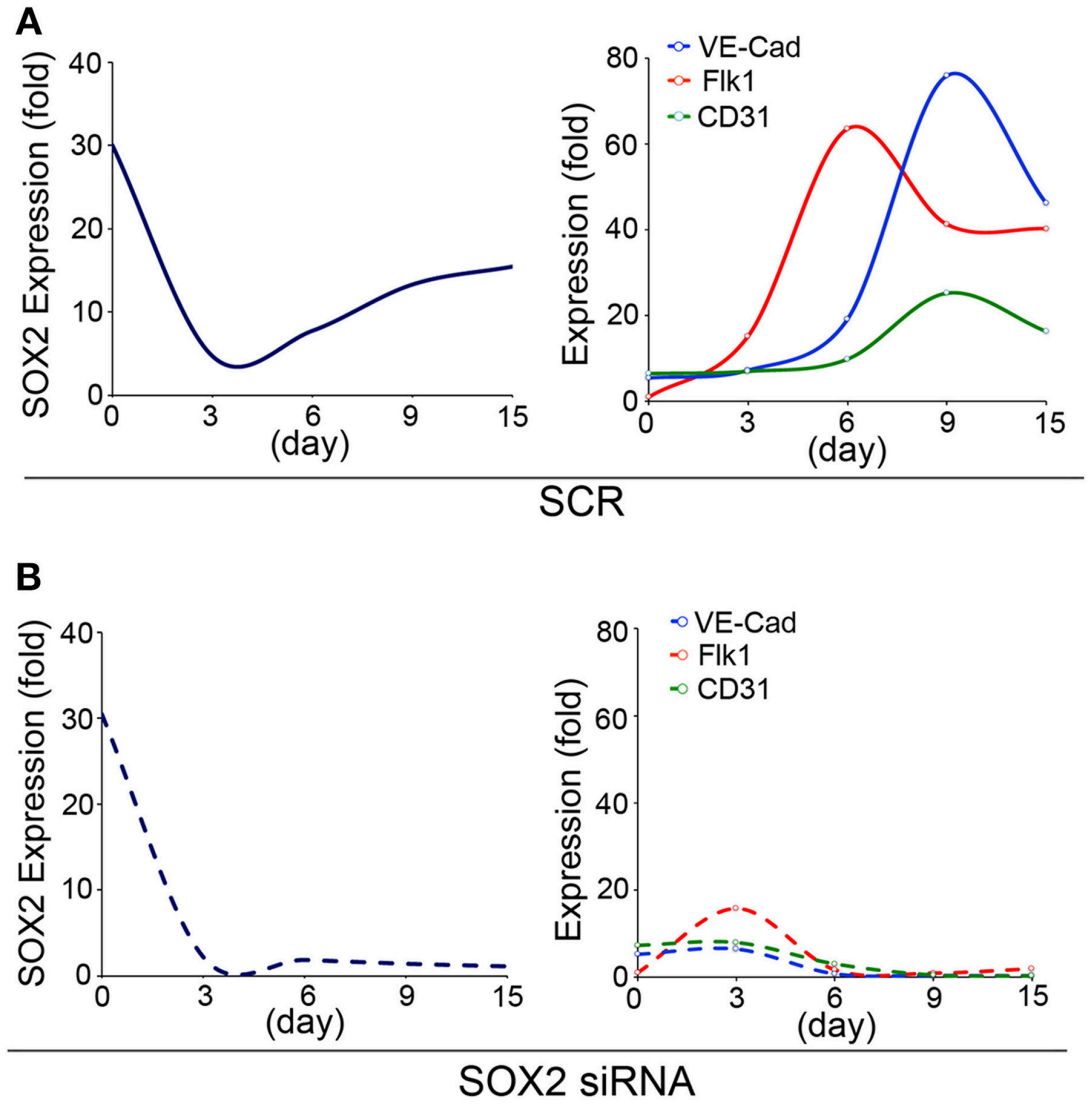
SOX2 plays a role in endothelial differentiation. Time course of expression of (left panels) SOX2, and (right panels) fetal liver kinase 1 (Flk1), VE-cadherin (VE-Cad) and cluster of differentiation 31 (CD31), as determined by real-time PCR during endothelial cell derivation from wild type embryonic stem cells (15 days). **(A)** Scrambled siRNA (SCR) or **(B)** specific *Sox2* siRNAs were transfected into the embryonic stem cells on day 3. Gene expression is shown as fold change compared to the expression on day 0 (*n* = 5).

To study the role of SOX2 in the developing endothelium, we used an embryonic stem cells (ESCs) model of endothelial differentiation (48) and examined the temporal expression of SOX2 and endothelial markers. The endothelial markers emerged between day 3 and 6 of endothelial induction, as the expression of SOX2 increased (Figure 1A), which suggested that involvement of SOX2 might be required for EC differentiation. Therefore, we depleted *Sox2* transcripts in the ESCs on day 3 using siRNA, and found that the reduction of SOX2 suppressed EC differentiation (Figure 1B). Interestingly, the cells still kept the ability to differentiate into other lineages after depletion of *Sox2*, including neuronal differentiation (data not shown), suggesting that suppression of *Sox2* may alter the direction of endothelial differentiation. Our results supported a role for SOX2 in the endothelial integrity, although it is unknown if SOX2 directly targets or interacts with early drivers of endothelial differentiation.

SOX2 is also known to be a key regulator of neuronal differentiation (37). In previous studies, we found ECs that were double positive for the endothelial marker fetal liver kinase 1 (Flk1) and SOX2 adjacent to differentiating brain cells on E10.5 and E14 (49). Flow cytometric analysis of dissected E12.5 embryonic brains confirmed the presence of cerebral ECs that co-expressed the endothelial marker VE-cadherin and SOX2 (49). This suggested that the ECs and the brain cells originated from the same progenitor cells and the endothelial and neuronal differentiation were coordinated. Similar observations were made in other organs, such as the lungs and the liver (49), suggesting the presence of differentiation “forks” involving ECs and organ-specific cells that are aimed at coordinating the developmental progression.

**Figure 2:**
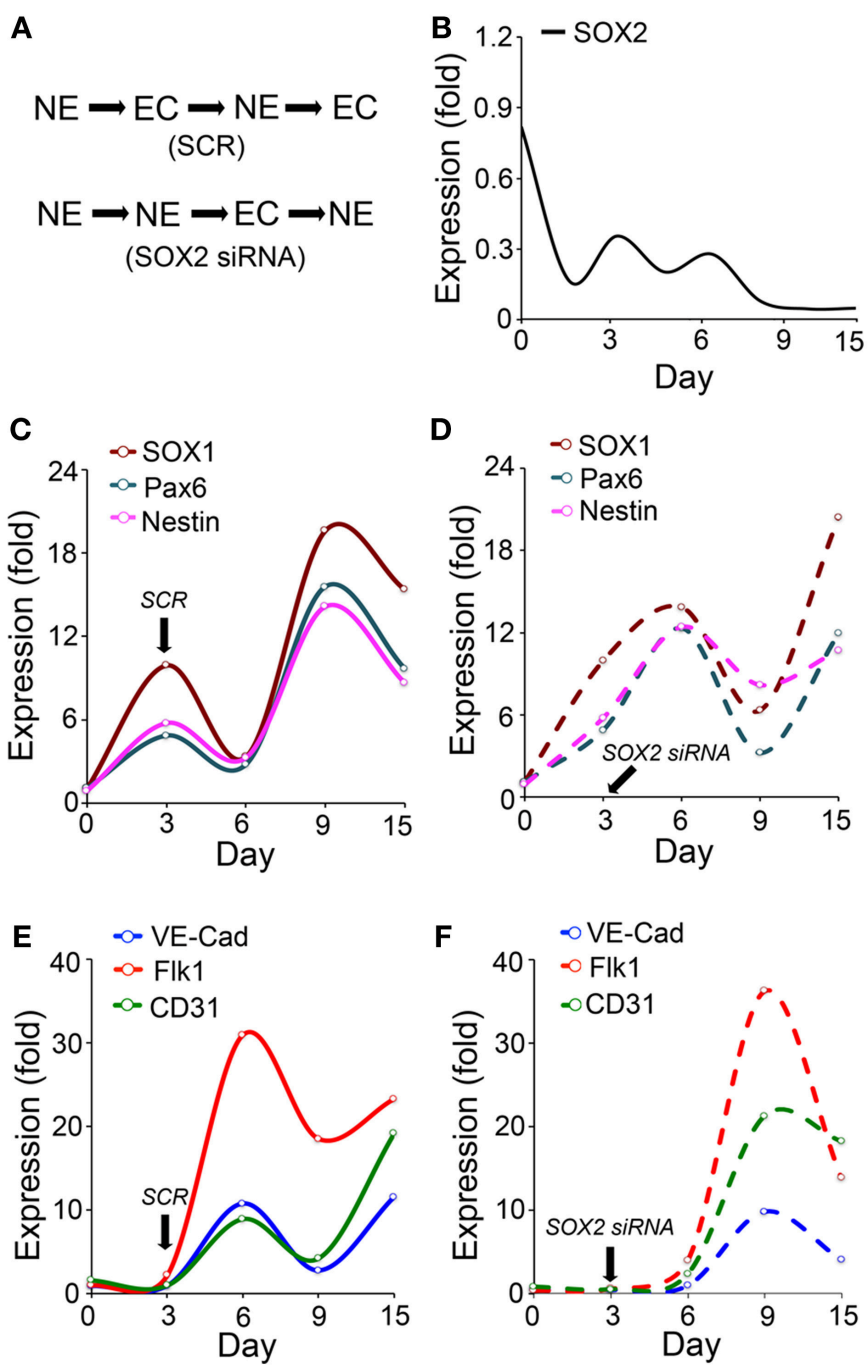
Endothelial differentiation co-exists with neural differentiation. **(A)** Schematic diagram of the temporal induction patterns of coordinated endothelial (EC) and neuronal (NE) differentiation. **(B–F)** Marker expression during neuronal differentiation in wild type embryonic stem cells with or without suppression of SOX2 (*n* = 5). Scrambled siRNA (SCR) or specific *Sox2* siRNAs were transfected into the cells on day 3. Expression was determined by real-time PCR and calculated as fold change compared to the expression on day 0. **(B–D)** Time course of expression of SOX2, and the neural progenitor markers SOX1, paired box protein (Pax6), and nestin. **(E,F)** Time course expression of the endothelial markers VE-cadherin (VE-Cad), fetal liver kinase 1 (Flk1) and cluster of differentiation 31 (CD31).

To further examine the potential role of SOX2 in such a coordination, we induced neuronal differentiation in ESCs (50). We found that the *Sox2* depletion delayed the endothelial marker induction and changed the temporal sequence of neuronal and endothelial differentiation (Figure 2A). The results showed that expression of SOX2 peaked twice during the neuronal differentiation, on day 3 and day 6 (Figure 2B), which differs from the SOX2 expression in endothelial differentiation (Figure 1). We examined the expression of the neuronal lineage markers SOX1, paired box protein (Pax6), and Nestin, together with the endothelial markers VE-cadherin, Flk1, and cluster of differentiation 31 (CD31) for up to 15 days. The results showed that the endothelial markers were induced during neuronal differentiation even without specific EC induction (Figures 2C–F). The time course suggested that the two types of differentiation were orchestrated, such that expression of endothelial markers was high when expression of neuronal markers was low, and vice versa (Figures 2C–F). The temporal sequence in this cell model appeared to be neuronal-endothelial-neuronal-endothelial (Figure 2A).

## SOX2, EndMTs, and Vascular Calcification

Vascular calcification is a frequent complication of vascular disease (7, 51–54) that exhibits multiple patterns of calcification depending on the type of disease, the type of vessel and the vascular layer that is affected (55). Several sources of calcifying cells have been identified, including vascular medial cells such as smooth muscle cells and pericytes, adventitial cells, ECs, various progenitor cells and osteoclast-like cells (55). Reports of endothelial contributions to calcific lesions suggest that EndMTs mediate direct contributions of osteogenic cells from the endothelium, thereby giving the endothelium a direct role in the development of vascular calcification.

EndMTs occur first in development and have been clearly demonstrated during heart valve formation (8, 56, 57) and recur in adult disease processes in the cardiac valves (8, 58), pulmonary artery hypertension (59), atherosclerosis and vascular calcification (59, 60). Several members of the transforming growth factor (TGF)beta superfamily, such as TGFbeta and bone morphogenetic proteins (BMPs) (61) have been shown to be important regulators of EndMTs.

Abnormal TGFbeta signaling induces mesenchymal-like phenotype in a variety of ECs (8, 59, 62–64) and both BMP4 and BMP6 have been implicated in EndMTs (65–67). Mutations in the BMP receptor activin receptor-like kinase 2 (ALK2) are causative in fibrodysplasia ossificans progressiva, where capillary ECs contribute cells to calcific lesions through endothelial transitions (13). Gene deletion of the BMP inhibitor matrix Gla protein (MGP) results in excess BMP signaling and rapidly developing arterial calcification (68) involving extensive EndMTs (10, 65).

Several factors have emerged as important participants in the crosstalk between TGFbeta and BMP signaling in EndMTs. These include Notch signaling, which is essential in EndMTs in heart development and valve formation [reviewed in (59, 69)], hypoxia (70) and fibroblast growth factor (FGF) signaling (71). Another factor is Wnt signaling, which is active in lymphatic ECs responding to Wnt5b (72), valvular ECs responding to matrix stiffness (73), and ECs transitioning to cardiac smooth muscle cells and pericytes under the influence of paracrine Wnt ligands (74). Interestingly, lack of primary cilia in a model of mouse embryonic ECs has been shown to increase the propensity to undergo EndMTs and osteogenesis in response to BMP signaling (75), potentially due to altered responses to mechanical and chemical stimuli.

**Figure 3:**
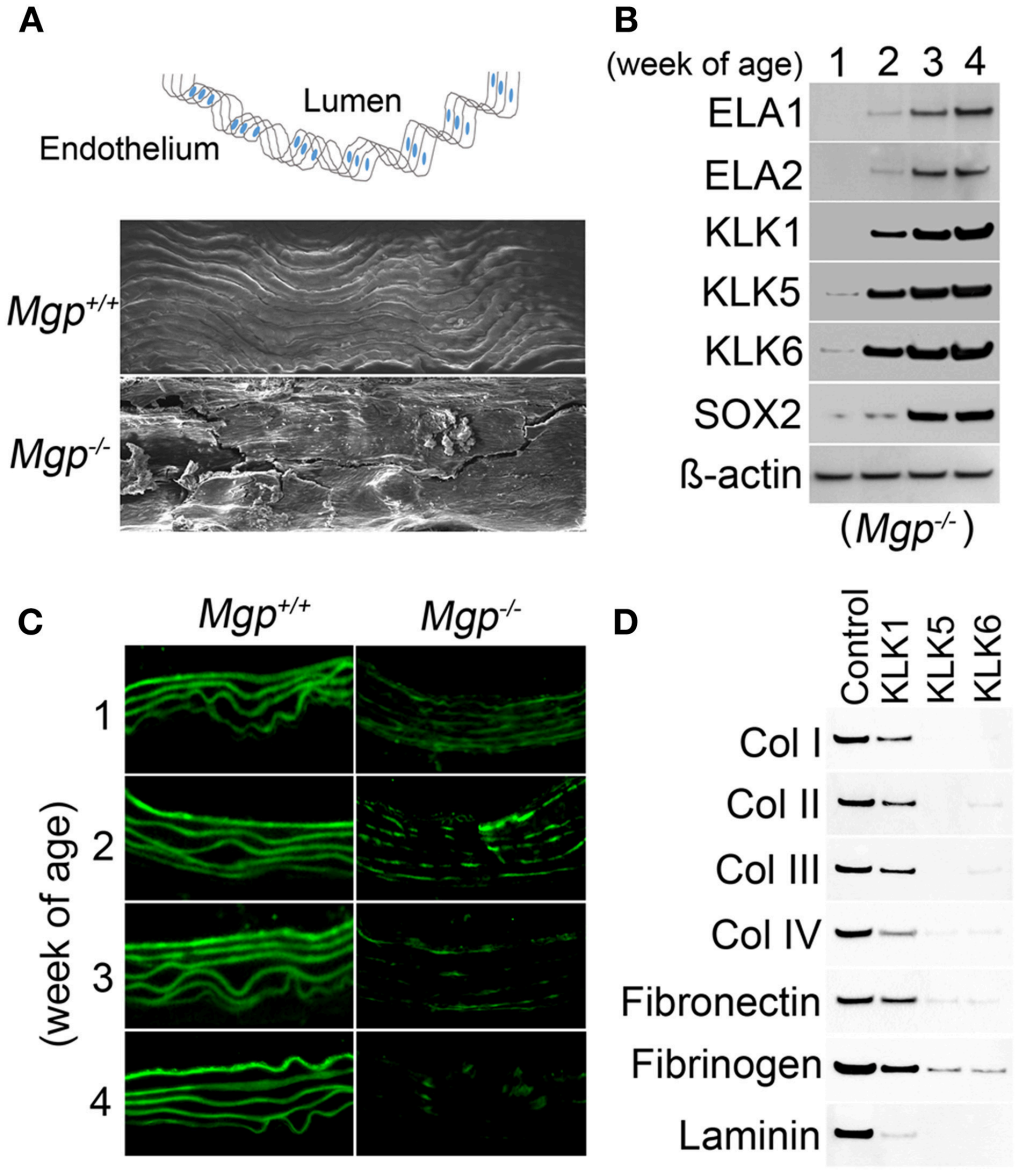
Serine proteases degrade Mgp-/- aortic tissue **(A)** Wild type (*Mgp*^+/+^) and *Mgp*^−/−^ aortic endothelium at 4 weeks of age was examined by scanning electron microscopy (magnification 5 × 10^2^). The image shows significant disruption of the *Mgp*^−/−^ endothelium and elastic lamina (*n* = 3). Top, schematic diagram indicating the locations of lumen and endothelium. **(B)** Expression of elastase (ELA) 1 and 2 and kallikrein (KLK) 1, 5, and 6 and SOX2 in *Mgp*^−/−^ aortas at 1–4 weeks of age, as determined by immunoblotting. Beta-actin was used as a loading control (*n* = 3). **(C)** Immunofluorescent staining of aortic elastin of wild type (*Mgp*^+/+^) and *Mgp*^−/−^ mice at 1–4 weeks of age. The images reveal the degradation of *Mgp*^−/−^ aortic elastin starting at 2 weeks of age (*n* = 5). **(D)** Immunoblotting demonstrates that kallikrein (KLK) 1, 5, and 6 degrade matrix components of aortic tissues including collagen (Col) 1, II, III, and IV, fibronectin, fibrinogen and laminin *in vitro*. One mg of substrate protein was mixed with 100 ng of enzyme or control at 37°C for 1 h before the samples were analyzed by immunoblotting (*n* = 3).

**Figure 4:**
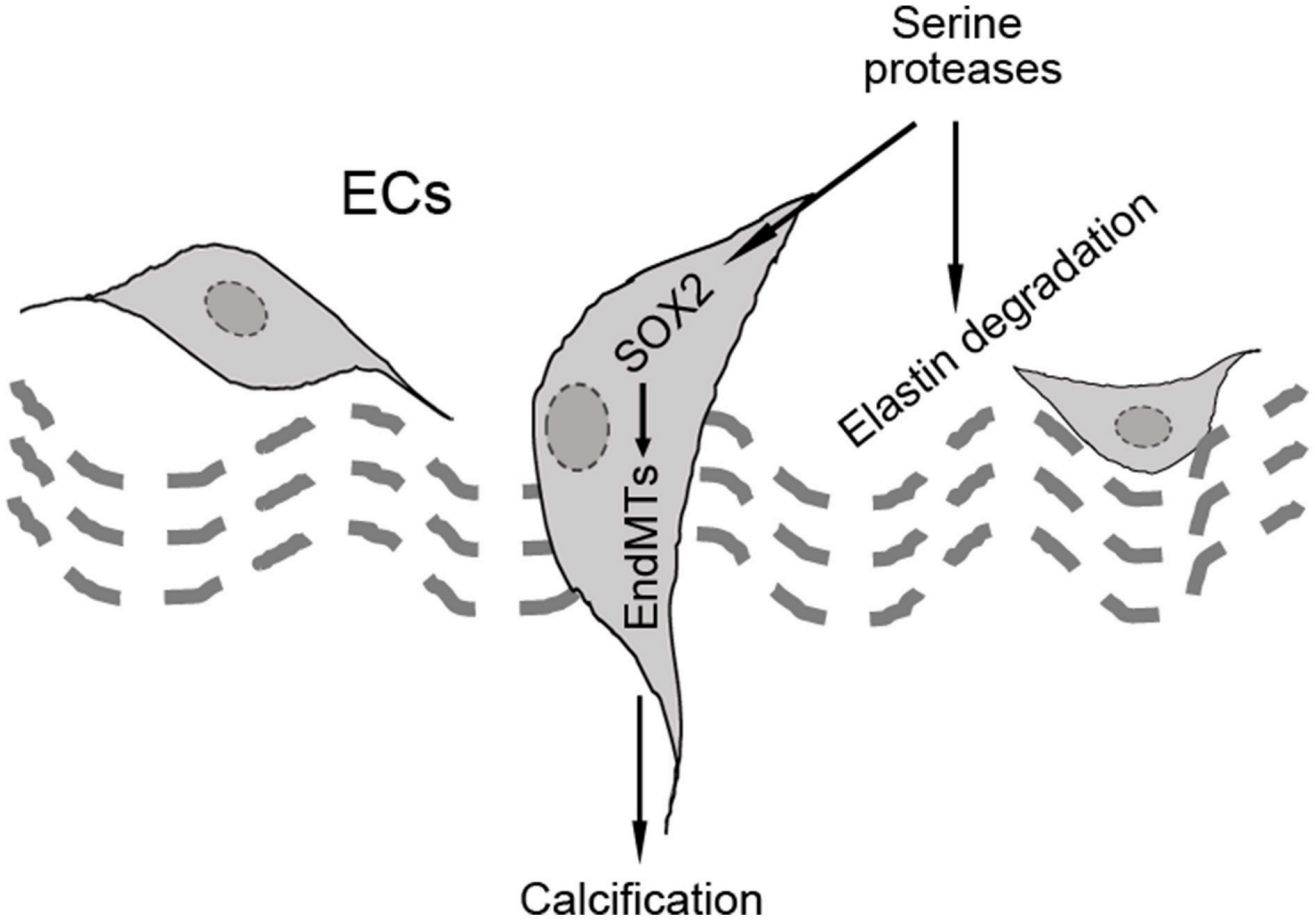
Schematic diagram of the hypothesis that SOX2 acts as a mediator of endothelial-mesenchymal transitions (EndMTs).

In our studies using the *Mgp*^−/−^ and *Ins2*^Akita/+^ diabetic mouse models of vascular calcification, we identified SOX2 as a response gene to ectopic BMP activity and a master regulator of EndMTs (10, 65). EndMTs are especially prominent in the aortas of *Mgp*^−/−^ mice (10–12, 65), where they contribute to the rapid calcification. Observed through phase contrast and transmission electron microscopy, the endothelium was highly abnormal with a mixture of cells largely replacing normal ECs, including chondroblast-like cells (65). EC-like cells were surrounded by abnormal matrix and detached from the internal elastic lamina (IEL). Transmission and scanning electron microscopy showed a marked degradation of the IEL, usually in close contact with endothelium [(65), Figure 3A]. Ultimately, the IEL became undetectable with the EC-like cells positioned as if migrating from the luminal side toward the calcifying lesions (65). Endothelial markers were detected deep in the calcified media, where they co-localized with osteogenic markers (65). Further studies revealed that the degradation of the IEL resulted from the induction of a complex of specific serine proteases including elastase 1 and 2 and kallikrein 1, 5, and 6 (65). The expression of these serine proteases increased dramatically in association with the degradation of both the IEL and the elastic lamellae in the media and induction of endothelial SOX2 as the *Mgp*^−/^^−^ aortas calcified (Figures 3B,C). Assessment of the proteolytic activity showed that the proteases are able to degrade collagen I, II, III, and IV, fibronectin, fibrinogen, and laminin (Figure 3D). In addition, the serine proteases were able to induce endothelial SOX2 and activate EndMTs (65). Both serine protease inhibition and *Sox2* depletion in the endothelium diminished EndMTs and vascular calcification *in vitro* and *in vivo* (65). Similar results for SOX2 as a mediator of vascular calcification were also found in atherosclerotic and diabetic mice, which showed that genetically limiting SOX2 in *Ins*^*Akita*/+^ mice or inhibiting SOX2 by siRNA in *Apoe*^−/^^−^ mice fed a Western diet reduced vascular calcification (11, 12). We argue that on one hand, the induction of the serine proteases plays an initial role in triggering endothelial SOX2 and activating EndMTs. On other hand, the local milieu with excessive degradation of elastin and cell-matrix allows the transitioning ECs to migrate and contribute to the calcification (Figure 4).

Potential roles for other SOX transcription factors in acquired vascular disease have not been well-studied. SOX17 has been identified as a risk locus for intracranial aneurysms (76) and SOX17 deficiency, which affects EC regeneration, may predispose to stress-induced intracranial aneurysms in hypertensive mice (77). SOX18 expression has been reported in advanced coronary atherosclerotic lesions (78), where it is expressed in both ECs and smooth muscle cells and may be involved in cell growth. However, it is unknown if SOX2 interacts with other members of SOX family to induce EndMTs.

Altogether, the SOX transcription factors are emerging as increasingly important players in cellular transitions, endothelial dysfunction and vascular disease. The SOX factors may be useful targets for endothelial modulation in the prevention or treatment of vascular calcification.

## Methods

### Animals

*Mgp*^+/−^ (B6.129S7-Mgptm1Kry/KbosJ) on C57BL/6J background were obtained from the Jackson Laboratory. Genotypes were confirmed by PCR (79), and experiments were performed with generations F4-F6. Littermates were used as wild type controls. All mice were fed a standard chow diet (Diet 8604, HarlanTeklad Laboratory). The studies were reviewed and approved by the Institutional Review Board and conducted in accordance with the animal care guidelines set by the University of California, Los Angeles. The investigation conformed to the National Research Council, *Guide for the Care and Use of Laboratory Animals, Eighth Edition* (Washington, DC: The National Academies Press, 2011).

### Tissue Culture and Cell Differentiation

Wild type ESCs (C57BL/6J background) were obtained from American Type Culture Collection (ATCC) (SCRC-1002). Mouse ESCs were cultured and maintained as previously described (48, 80). The derivation of endothelial cell differentiation from ESCs was performed using previously published protocols (48, 81). BMP-4, Activin A, FGF-2, and VEGF (all from R&D Systems) were added to StemPro-34® medium prior to use. The process of derivation lasted 14 days.

Neuronal differentiation in ESCs was performed using previously published protocols (82). Briefly, mouse ESCs without feeder cells were dispersed into a single cell suspension with 0.25% trypsin. Aggregation of ESCs was induced by preparing hanging drops of medium (20 μL) on the lids of petri dishes (2000 cells per drop) on day 0. After 2 days, embryonic bodies were harvested in petri dishes, where they matured for 3 days. The medium was changed every 1–2 days based on the number of dead cells. On day 5, the embryonic bodies were plated on coated cell culture dishes. In order to increase the adherence of embryonic bodies, the dishes were coated with laminin for one day and D-lysine for another day before use. The embryonic bodies were subsequently cultured for two more days. The medium was changed on day 7, and every 1–2 days after that. Mature neurons were observed subsequently to day 7.

### Protease Assay

Kallikrein 1, 5, and 6 (all 10 ng/mL; Abnova) were individually added to the purified proteins, collagen I, II, III, and IV, fibronectin, fibrinogen, and laminin, and incubated at 37°C for 1 h. After the incubation, the mixtures were examined by immunoblotting to assess the degradation of each protein using specific antibodies. The carrier was used as a control.

### RNA Analysis

Real-time PCR analysis was performed as previously described (65). Glyceraldehyde 3-phosphate dehydrogenase (GAPDH) was used as a control gene (65). Primers and probes for mouse SOX2, Flk1, CD31, VE-cadherin, SOX2, Pax6, and Nestin were obtained from Applied Biosystems as part of Taqman® Gene Expression Assays.

### Immunofluorescence

Immunofluorescence was performed as previously described (65) with specific antibodies for elastin (Abcam). The nuclei were stained with 4',6-diamidino-2-phenylindole (DAPI, Sigma-Aldrich).

### Scanning Electron Microscopy

Aortic tissue samples were analyzed by scanning electron microscopy as previously described (65).

### Immunoblotting

Immunoblotting and immunoprecipitation were performed as previously described (65). Equal amounts of tissue lysates were analyzed, and the blots were incubated with specific antibodies to elastase 1 (Sigma-Aldrich), elastase 2 (Abgent), kallikrein 1 (Sigma-Aldrich), kallikrein 2 (Abgent), kallikrein 5 (Acris Antibodies), kallikrein 6 (Sigma-Aldrich), and collagen I, II, III, IV, fibronectin, fibrinogen and laminin (all from Abcam) as previously described (65). Beta-Actin (Sigma-Aldrich) was used as a loading control.

### Statistical Analysis

Data were analyzed for statistical significance by ANOVA with *post-hoc* Tukey's analysis. The analyses were performed using GraphPad Instat®, version 3.0 (GraphPad Software). Experiments were repeated a minimum of three times.

## Author Contributions

YY and KB supervised the experiments, analyzed data, and wrote the manuscript. JY performed experiments and data analysis.

### Conflict of Interest Statement

The authors declare that the research was conducted in the absence of any commercial or financial relationships that could be construed as a potential conflict of interest.
